# Randomized Pilot Trial of Pre‐ and Postoperative Heart Failure Nurse‐Supported Care in Heart Failure Patients Requiring Noncardiac Surgery—Feasibility and Results

**DOI:** 10.1002/clc.24304

**Published:** 2024-06-24

**Authors:** Ester J. Herrmann, Badrinarayanan Raghavan, Meaza Tekeste, Kathleen Mantzsch, Patrick Meybohm, Birgit Assmus

**Affiliations:** ^1^ Department of Cardiology and Angiology University Hospital Giessen and Marburg Giessen Germany; ^2^ Department of Cardiology University Hospital Frankfurt/Main Giessen Germany; ^3^ Department of Anesthesiology, Intensive Care and Emergency and Pain Medicine University Hospital Würzburg Würzburg Germany

**Keywords:** elective noncardiac surgery, heart failure, nurse‐based HF care, preoperative HF screening

## Abstract

**Introduction:**

The perioperative cardiovascular management of patients undergoing noncardiac surgery is particularly challenging in those with pre‐existing heart failure (HF). This study was designed to evaluate the effectiveness of nurse‐based pre‐ and postoperative specialized HF management in reducing postoperative HF‐associated complications in patients with known HF undergoing noncardiac surgery.

**Methods:**

This prospective, randomized pilot study included patients with established HF requiring intermediate‐ to high‐risk noncardiac surgery. Patients received postoperatively either standard care (control group, CG) or nurse‐supported HF management (intervention group, IG). The primary endpoint was a composite of HF‐related postoperative complications at 30 days. Secondary endpoints included length on intensive care unit, length of hospital stay, death, hospitalization for HF, and quality of life assessment using the SF‐12 questionnaire.

**Results:**

The trial was halted prematurely for futility. A total of 34 patients (median age 70.5 [IQR 67–75] years; with 15 HfpEF, 9 HfmrEF,10 HfrEF), with an average NT‐proBNP of 1.413 [463–2.832] pg/mL were included. The IG had a lower rate of postoperative primary events (25%; *n* = 4) compared with the CG (33%; *n* = 6). There were no differences in secondary endpoints between the groups. Quality‐of‐life scores improved slightly in both groups (*δ* 5.6 ± 0.9 [CG] and 3.1 ± 1.2 [IG]).

**Conclusion:**

Nurse‐based pre‐ and postoperative HF care appears to be feasible and may reduce HF‐associated complications in patients undergoing noncardiac surgery. Larger clinical trials are needed to further evaluate the effectiveness of this approach in reducing postoperative complications in this high‐risk patient population.

## 1 Introduction

The cardiovascular management of patients with elective and urgent noncardiac surgery is increasingly challenging due to the demographic ageing of the population and its growing comorbidity burden [1]. The risk of perioperative complications depends on the functional status of the patient before surgery, the prevalence of comorbidities, and the degree of urgency, type, extent, and duration of the surgical procedure. In particular, cardiovascular and cardiac complications occur more frequently in association with established heart failure (HF) or in patients who suffer from asymptomatic ischemic heart disease, left ventricular dysfunction, valvular heart disease, or arrhythmias undergoing surgical procedures with considerable hemodynamic and cardiac stress [2]. Systematically gathered data concerning the annual numbers of surgical procedures and the associated complication rates for the EU or Germany are not available [3]. Smilowitz et al. revealed that severe cardiovascular and cerebrovascular perioperative complications occur in 1 of 33 hospitalizations for noncardiac surgery in the United States [4].

A modeling analysis, based on global data from 2004, estimated the rate of surgical procedures with a high procedure‐associated risk to be about 4% of the world population per year [3]. If this rate were applied to Europe with a population about 500 million people, it would result in about 19 million procedures with a high procedure‐associated risk per year. Even if the majority of these procedures were conducted with minimal cardiovascular risk, nearly half of adults aged ≥ 45 years undergoing major noncardiac surgery present with at least two cardiovascular risk factors [5], and 30% of these patients suffer from a significant cardiovascular comorbidity. Thus, an estimated 5.7 million procedures per year are performed in patients with considerable risk for cardiovascular complications.

The average cardiovascular complication rate of noncardiac surgery is estimated to be between 7% and 11%, with a mortality rate of 0.8%–1.5% [6]. In 42% of deaths, a cardiac complication is considered to be the primary cause of death [7], and a US analysis from 2008 showed that 18% of the patients >65 year of age undergoing noncardiac surgery suffer from HF [8]. Given the ageing of the population of western societies, this presents an increasing challenge for the perioperative patient management [9]. Current estimations suggest that older people need four times more surgical procedures compared than the younger population [10, 11]. Thus, it is assumed that the number of patients undergoing an elective surgical procedure will increase at least by 25% until 2030 in Europe, with 20% of patients being >75 old [1].

In Germany, up to three million patients suffer from chronic HF, which is the most frequent cardiovascular disease due to its age‐associated increase in prevalence [12]. More than 50.000 deaths per year can be attributed to chronic HF [13].

HF nurses are not yet routinely involved in out‐ or inpatient care in Germany, although the benefit of multidisciplinary HF care programs including specialist nurses has been demonstrated by various authors and is recommended for outpatient management by current ESC guidelines [14].

Given the increasing prevalence of HF in elderly patients requiring noncardiac surgery and their HF‐associated with increased morbidity and mortality risks, the aim of the present study was to evaluate whether HF nurse‐based pre‐ and postoperative specialized HF management, in comparison to standard care, has the potential to reduce postoperative HF‐associated complications in patients with established HF undergoing an inpatient noncardiac surgical procedure.

## 2 Methods

### 2.1 Patient Population and Study Design

This is a prospective, randomized, open‐label, interventional pilot study performed at a single center from October 2017 until September 2019 that compared standard care (control group) with nurse‐based HF management (intervention group) in a noncardiac surgical cohort. Patients with established or suspected HF (either with HF with reduced ejection fraction, HFrEF [LVEF ≤ 40%], or HF with mildly reduced or preserved ejection fraction, HFmrEF/HFpEF [LVEF > 40%] and at least NYHA class II or elevated levels of N‐terminal fragment of pro‐brain natriuretic peptide, NT‐proBNP) undergoing an elective inpatient noncardiac surgical procedure of intermediate or high risk according to ESC risk classification [2, 15] with at least 24 h in hospital, were screened for inclusion into the trial. Further inclusion criteria were age ≥ 18 years and ability to give informed consent. Exclusion criteria were preoperative therapy on intensive care unit, end‐stage kidney disease requiring dialysis, or a life expectancy <3 months. For screening, patients were referred from anesthesiology premedication clinics to cardiology for further evaluation.

Immediately after they provided written informed consent, patients were visited by a HF nurse. If advised by the cardiology team and upon agreement by the surgical team, a preoperative optimization of HF therapy or further diagnostics was performed, and surgery was postponed in these cases. 1:1 randomization to either standard care or HF groups occurred after optimization and immediately within the electronic case report form (secuTrial, interActive Systems GmbH, Berlin, Germany) before surgery.

Standard care (control group) was defined as routine care and included of obtaining additional HF counseling upon request at any time. Study procedures within the nurse‐based HF postoperative management (intervention group) comprised regular nurse visits that began the day after surgery during working days and continued at least every other day until discharge. These visits included a prespecified HF‐specific approach with evaluation of physical parameters (including blood pressure, heart rate, edema assessment, respiration frequency, current fluid balance, check of HF medication assessment, and kidney function parameters), as well as pain level and mood assessment. Advice for treatment changes was provided to the responsible surgeon on the ward after consultation with the cardiologist in charge. In addition, at least one postoperative cardiologist visit was performed together with the nurse.

For postoperative follow‐up, there were scheduled contacts by telephone at 30 days, and by outpatient visit or telephone at 3 months.

### 2.2 Study Endpoints

The primary endpoint was a combined endpoint consisting of the following events up to 30 days after surgery: readmission to intensive care unit, need for inotropic therapy, pleural effusion, X‐ray‐confirmed pulmonary edema, antibiotic treatment for pneumonia, and ventilation (noninvasive and/or invasive).

Secondary endpoints were duration on the intensive or intermediate care unit and of the hospital stay at 30 days, duration of total hospital stay at 90 days, number of pleural punctures, number of pulmonary edema events, days of (non)‐invasive ventilation, days of antibiotic treatment for pneumonia, days of inotropic therapy at 30 days, acute kidney injury (acute kidney injury defined as Stage 1 = creatinine increase > 1.5–1.9 × baseline, Stage 2 = creatinine increase > 2–2.9 × baseline, Stage 3 = creatinine increase > 3 × baseline, dialysis) [16] at 30 and 90 days, number of diuretic dosage adjustments, heart rhythm disorders (new onset of atrial fibrillation, life‐threatening ventricular tachycardia, implantation of pacemaker due to bradycardia) at 30 days, NT‐proBNP and high‐sensitivity troponin T at admission versus at 30 days follow‐up and discharge, mortality, total rehospitalization and rehospitalization for HF at 30 days, and quality of life (SF‐12 questionnaire) at 30 and 90 days.

### 2.3 Quality‐of‐Life Questionnaire

Quality of life was assessed using the SF‐12 score before surgery and at the 30 days and 3 months follow‐up. Each item of the SF‐12 was scored on a 5‐point scale. Each scale score was then calculated as the mean of its item scores and transformed to a 0–100 scale using the following formula, with higher scores indicating higher levels of functioning:

$$\mathrm{Transformed}\;\mathrm{Scale}=\left[\frac{\left(\mathrm{Actual}\;\mathrm{raw}\;\mathrm{score}-\mathrm{lowest}\;\mathrm{possible}\;\mathrm{raw}\;\mathrm{score}\right)}{\mathrm{Possible}\;\mathrm{raw}\;\mathrm{score}\;\mathrm{range}}\right]\times 100.$$

### 2.4 Sample Size Calculation

Initially, we aimed to include 525 patients into the trial. These numbers were based on the following assumptions: (1.) the frequency of HF‐related complications at our hospital as well as in the German health care setting is unknown, but, according to the literature and after consultation with our colleagues in the anesthesiology department, we estimated that 25% of included patients at risk will experience at least one complication of the combined primary endpoint; (2.) we estimated that we can reduce these HF‐related complications to 15% (relative risk reduction 60%); (3.) given an *α* a of 0.05 and a power of 0.8 this would result in 250 patients per group (uncorrected *χ* ^2^ test); (4.) with a 10% lost‐to‐follow‐up rate, this would result in 525 patients in the trial. Given that in our hospital, more than 7500 noncardiac surgical procedures are performed per year, we anticipated that there would be enough patients with HF who could be recruited into our trial.

However, already during the early phase of the trial, we realized that identification of HF patients from patient history or clinical presentation in the routine setting of the anesthesiology clinic is difficult without additional procedures like NT‐proBNP testing. Thus, among 346 referrals with a high additional cardiological workload, there were only 72 patients with confirmed HF, and the final cohort comprised only 36 patients enrolled within 18 months. Therefore, the trial was halted prematurely for futility.

### 2.5 Statistics

The present study represents a descriptive analysis of two patient cohorts. Baseline characteristics are shown as median and interquartile range. Quality‐of‐life analysis (SF‐12) was carried out using the overall summary score. Because of the premature study termination, we made only a statistical comparison of the primary combined endpoint by using the Mann–Whitney *U*‐test, after exclusion of normal distribution by the Shapiro–Wilk test. In addition, we analyzed receiver‐operating characteristics curves for baseline NT‐proBNP levels in patients with and without a primary endpoint event. All analyses were carried out using SPSS (Version 29.0.1.1, SPSS Inc., IBM Corp., Armonk, NY, USA) and graphs were done using GraphPad Prism software (version 9.0) for MS Windows (GraphPad Software, San Diego, CA, USA).

## 3 Results

**Figure 1:**
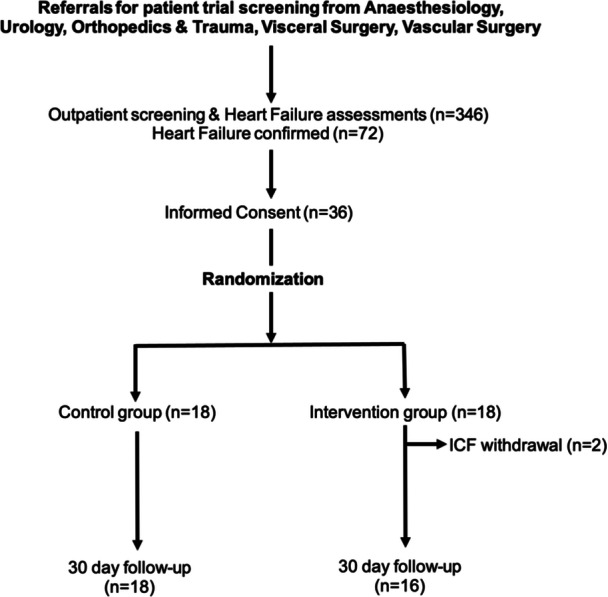
Design of the patient recruitment: Recruitment of trial subjects based on inclusion and exclusion criteria.

A total of 346 patients were referred from anesthesiology and screened during the 1.5‐year enrollment period. HF was confirmed in 72 patients, and 36 patients were finally included and randomized. However, two patients from the intervention group withdrew their consent and were therefore not available for the present analysis. In addition, the surgical procedure was canceled after randomization in one patient. One patient died at 16 days postoperatively due to pulmonary embolism and one patient was lost to follow‐up at 30 days (Figure 1). Unfortunately, only 22 patients (64.7%) could be contacted at 90 days (13 by telephone contact and 9 during clinical visits), resulting in a rate of 35% lost to follow‐up. The present manuscript thus focuses on the 30‐day outcomes of the control group (18 patients) and the intervention group (16 patients), and 90‐day results are shown in Supporting Information S1: Table S1 and Figures S1A,B.

The final cohort was predominantly male, elderly HF patients, with 15 patients suffering from HFpEF, 9 from HFmrEF, and 10 from HFrEF. Before the surgical procedure, median NT‐proBNP was profoundly elevated (1413 [IQR 463–2832] pg/mL), corresponding to a median functional NYHA class of 2 [2, 3]. Systolic blood pressure was high‐normal (130 [110–142] mmHg) (Table 1). In the intervention group, numerically more patients were treated with renin‐angiotensin‐aldosterone system (RAAS) inhibitors (81 vs. 78%), mineralocorticoid receptor antagonists (MRA) (44 vs. 22%), and diuretics (94% vs. 78%) and fewer with beta‐blockers (75% vs. 94%) compared with patients in the control group (Supporting Information S1: Table S2).

**Table 1:** Baseline characteristics of control and intervention groups.

	Control group (*n* = 18)	Intervention group (*n* = 16)
Age (median, IQR, years)	70.5 (69–75)	70 (65–74)
Sex, *n* (%)		
Male	5 (27.8)	4 (25)
Female	13 (72.2)	12 (75)
Type of surgery, *n* (%)		
Vascular surgery	8 (44.4)	7 (43.8)
Abdominal surgery	3 (16.7)	2 (12.5)
Trauma and orthopedic surgery	4 (22.2)	2 (12.5)
Urological surgery	3 (16.7)	5 (31.3)
BMI (kg/m^2^), median (IQR)	26.6 (23.6–31.3)	27.7 (26.2–33.4)
Hypertension, *n* (%)	18 (100)	16 (100)
Active smoker, *n* (%)	1 (5.6)	4 (25)
Former smoker, *n* (%)	15 (83.3)	9 (56.3)
COPD, *n* (%)	4 (22.2)	0 (0)
Diabetes mellitus, *n* (%)	7 (38.9)	6 (37.5)
Chronic kidney disease with eGFR < 60 mL/min, *n* (%)	8 (44.4)	5 (31.3)
Peripheral artery occlusive disease, *n* (%)	7 (38.9)	3 (18.8)
Previous stroke, *n* (%)	1 (5.6)	4 (25)
ICD, *n* (%)	5 (27.8)	4 (25)
CRT‐D, *n* (%)	1 (5.6)	1 (6.3)
ICM, *n* (%)	10 (55.6)	7 (43.8)
Previous myocardial infarction, *n* (%)	8 (44.4)	5 (31.3)
NYHA class, median (IQR)	2 (2–3)	2 (2–3)
LVEF (%), median (IQR)	49 (35–55)	45 (20–56)
Systolic blood pressure (mmHg), median (IQR)	125 (110–140)	130 (110–147)
Creatinine (mg/dL), median (IQR)	1.19 (0.95–1.84)	1.35 (1.15–1.68)
NT‐proBNP (pg/mL), median (IQR)	1438 (527–4304)	951 (433–2681)
hs‐Troponin T (pg/mL), median (IQR)	19 (13–42) (*n* = 15)	21 (12–32) (*n* = 10)
Quality of life acc. to SF‐12, median (IQR)	50 (50–56)	37.5 (25–53)

Abbreviations: BMI, body mass index; COPD, chronic obstructive pulmonary disease; CRT, cardiac resynchronization therapy; hs, high‐sensitivity; ICD, implantable cardiac device; ICM, ischemic cardiomyopathy; IQR, interquartile range; LVEF, left ventricular ejection fraction; NT‐proBNP, N‐terminal fragment of pro‐brain natriuretic peptide; NYHA, New York Heart Association.

With respect to type of elective intermediate‐ to high‐risk noncardiac surgery, 15 patients required vascular surgery, 5 had abdominal, 6 trauma or orthopedics, and 8 urological surgeries.

In the postoperative setting, a total of 88 HF nurse visits were performed in 14 patients during the in‐hospital stay within the intervention group.

The primary combined endpoint occurred in 6 patients (33%) in the control group and 4 patients (25%) the intervention group. The contributing individual events are listed in Table 2.

**Table 2:** Primary combined endpoint and corresponding events at 30‐day follow‐up.

	Control group	Intervention group
Primary composite endpoint, *n* (%)	6 (33.3)	4 (25)
Individual components of the primary endpoint, *n* (%)		
Readmission to ICU	0 (0)	0 (0)
Inotropic therapy	0 (0)	0 (0)
Pleural effusion	3 (16.7)	2 (12.5)
Radiographically confirmed pulmonary edema	0 (0)	0 (0)
Antibiotic therapy for of pneumonia	1 (5.6)	0 (0)
Ventilation (invasive/noninvasive)	2 (11)	2 (12.5)

Abbreviation: ICU, intensive care unit.

**Figure 2:**
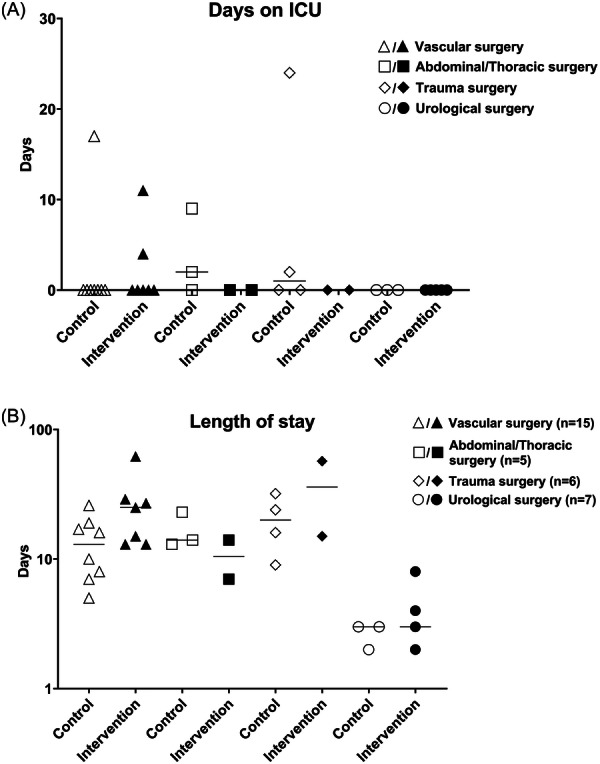
(A) Days on intensive care unit (ICU) compared for control and intervention group, by type of surgery. (B) Length of hospital stay for control and intervention group, by type of surgery.

With respect to the secondary endpoints, the median length of stay on the intensive care unit was 0 [0–1.5] days in the control group and 0 [0–0] days in the intervention group at 30 days (Figure 2A). The total length of hospital stay was 13.5 [7.3–18.5] days in the control group and 14 [7.5–26] days in the intervention group (Figure 2B).

Acute kidney injury was observed in one patient in the intervention group (AKI stage 1) and in three patients in the control group (once AKI stage 1 and twice AKI stage 2) (Supporting Information S1: Table S3).

**Figure 3:**
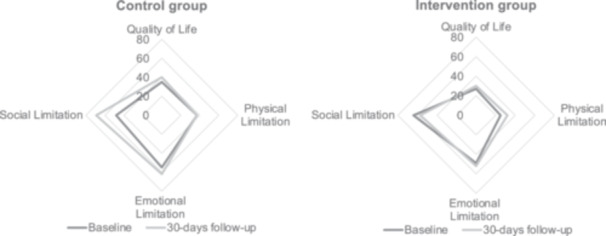
Quality of life assessments using the SF12 questionnaire. Quality of life within the different dimensions of the SF 12 and treatment groups comparing baseline to 30‐day follow‐up.

Quality‐of‐life analysis using the SF‐12 score (Supporting Information S1: Table S4), which was obtained from 34 patients at baseline and from 28 patients at the 30‐day follow‐up, revealed comparable impairment for the two groups before the surgical procedure (Supporting Information S1: Figure S2). There were moderate improvements at 30 days, more apparent with respect to social limitations, but less so regarding physical limitations (Figure 3). The results of SF‐12 and quality‐of‐life analysis for baseline, 30 days, and 90 days follow‐up assessments are shown in detail in Supporting Information S1: Tables S5 and S6.

Further exploratory endpoints at 30 days revealed numerically fewer HF‐related complications in the intervention group (Supporting Information S1: Table S3). In detail, two pleural punctures were carried out in the control group and there was no puncture in the intervention group. The number of days with de novo (non‐) invasive ventilatory support was low and was similar between the two groups, as were the number of days of antibiotic treatment for pneumonia. There was no need for re‐initiation of inotropic therapy in either group, whereas dose changes of diuretics were made 43 times in the control group and 17 times in the intervention group. In addition, de novo cardiac rhythm disorders occurred twice in the control group (new onset of atrial fibrillation and ventricular tachycardia) and once in the intervention group (new onset of atrial fibrillation, Supporting Information S1: Table S3).

Finally, we observed that the preoperative NT‐proBNP levels between patients with a composite primary endpoint event and those without were similar (1358 [483–4578] pg/mL) versus 1244 [492–2723] pg/mL, *p* = 0.3115).

## 4 Discussion

The present trial is the first pilot trial of HF nurse‐supported postoperative care in HF patients undergoing intermediate‐ to high‐risk noncardiac surgery in Germany. Although our results show that postoperative HF nurse supported care is a feasible approach in the German healthcare system, the major hurdle was to provide adequate screening and appropriate patient selection from surgical units for referral into such an interdisciplinary care trial. The fact that so few patients could be recruited into the present study demonstrates that the level of awareness of increased risk of postoperative complications in patients with HF, including HF with preserved ejection fraction, is low. For example, a routine biomarker screening approach, with NT‐proBNP testing in patients with clinical symptoms suggestive of HF under surgical or anesthesiological care is not yet widely established, although it is recommended in the latest ESC guidelines from 2022 [1]. Biomarker screening could thus be helpful to guide cardiology referral, which is currently performed at physician discretion and results in a high number of referrals without objective cardiac or HF problems.

However, once patients with confirmed HF were included into our trial, there was a 29.4% rate of HF‐related postoperative complications at 30 days, indicating that we selected a true high‐risk cohort. This is in line with the literature showing that cardiovascular complication rates vary between 10% and 20% after noncardiac surgery [17, 18]. However, these data are from different health care systems. Thus, no German data are available to estimate the number of HF‐related complications after surgical treatment. The most recent publication from Gualandro et al. reported a postoperative acute HF (pAHF) rate of 10% in chronic HF patients undergoing major noncardiac surgery and a pAHF rate of 1.5% in patients when HF was not pre‐existing [18]. Interestingly, HFpEF was the dominant phenotype of de novo acute decompensated HF. However, other complications like readmission to intensive care, requirement for ventilatory support, pleural effusion of pulmonary edema, or pneumonia have not been reported, although these complications can be triggered by HF and were therefore counted for the combined primary endpoint in our trial.

Importantly, reducing the number of HF‐related postsurgical complications should translate into a reduced length of stay, both on intensive and intermediate care units as well as in the hospital overall. Our pilot trial suggests that this may be feasible, but, obviously, more data are required. In addition, although the length of stay is mainly driven by medical needs, the outpatient environment is also a major determinant since especially elderly patients may require a geriatric rehabilitation program for which availability is limited. In detail, the length of hospital stay was 14 days in our cohort. Interestingly, in the Basel PMI group, the length of stay is between 7 days in the group without postoperative acute HF and 14 days in the group with postoperative HF [18]. However, the comparison is limited by the fact that postoperative HF was present in these cases, whereas in our cohort, all patients suffered from HF before randomization and rather developed postoperative complications due to aggravation of pre‐existing HF.

Previous analyses have demonstrated that adults aged ≥ 75 years have a greater rate of peri‐operative major adverse cardiovascular events (9.5% vs. 4.8% for younger adults *p* < 0.001) [19]. However, this is neither restricted to patients with pre‐existing HF nor to increased risk due to selection of intermediate‐ to high‐risk procedures. In our small cohort, the event rate was 29.4%, but the fact that our event definition was different, in addition to our selection of only elective intermediate‐ to high‐risk surgical procedures in HF patients, precludes any meaningful comparison.

Recent trials involving HF nurses have mostly focused on outpatient management, support, and telemedical care. Therefore, only a limited amount of data is currently available with respect to in‐hospital involvement of HF nurses or nurse practitioners (NPs). We identified a total of 4 trials investigating the additional impact of nurse‐supported care on patients with either acute decompensated HF or acute coronary syndrome in a cardiology ward. Two trials were randomized controlled trials investigating the impact of either inpatient support by an acute cardiac care NP in 1025 patients [20] or by a HF research nurse involved in transition (*n* = 48) to outpatient care [21]. Both trials reported a reduced 30‐days readmission rate. In addition, there are two reports of retrospective two‐group comparisons with a total of fewer than 500 patients that also report reduced HF readmission in the nurse‐supported care group [22, 23].

In the surgical setting, we identified a total of 4 trials in cardiac surgery patients with nurses or NPs involved during in‐hospital patient care. Among these, there are two randomized controlled trials including a total of 303 patients [24, 25]. However, primary endpoints were heterogenous, involving quality of life, patient satisfaction, symptoms, or healthcare use. In the observational studies [26, 27], acute cardiac care NPs were involved in patient management, which was observed to be associated with unchanged low mortality rates compared with historical controls but reduced costs.

We were not able to identify a single study of HF nurses or similar personnel involved in in‐hospital care of noncardiac surgery patients. Given that worldwide more than 200 million adults undergo noncardiac surgery annually, with an average overall complication rate of 7%–11% and a mortality rate of 0.8%–1.5% [6], we think future research with respect to this topic is urgently warranted.

Nurse‐based HF management may have the potential to reduce HF‐associated complications, which was shown numerically in our small pilot trial. However, whether the nurse‐based HF management may have any impact on the high postoperative mortality rate of 8% [28] needs to be investigated in further trials. Of note, perioperative mortality is greater in those with acute on chronic HF compared with chronic HF alone (7.8% vs. 3.9%) [29]. Therefore, the preoperative identification of HF patients and optimization of HF treatment before surgery might play a key role in prevention of HF‐specific postoperative complications. Such an approach could only be implemented by an interdisciplinary team that would tailor referral of patients at risk, with sufficient time until surgery, to a specific preoperative clinical cardiac risk assessment clinic. Biomarker testing is presently only recommended in patients at risk, according to current guidelines.

The recently initiated “PeriOP‐CARE HF” project, supported by the “GBA Innovationsfonds,” includes a multicenter randomized controlled multicenter trial to test a multifactorial intervention in 1094 patients >65 years old with elevated natriuretic peptides undergoing an elective, intermediate‐ to high‐risk noncardiac surgical procedure. The German Federal Joint Committee (G‐BA) is the highest decision‐making body representing physicians, dentists, hospitals, and health insurance funds, as part of a conditional coverage program in Germany for health‐care‐related costs. In this trial, patients undergoing a detailed preoperative in‐hospital risk assessment by an interdisciplinary team, which will be followed by goal‐directed pre‐ and perioperative care, and structured postoperative care, and transitional care to outpatient services supported by HF nurses, will be compared with patients with standard treatment. Recruitment will begin in 2024.

## 5 Limitations

The major limitations of the present analysis are the small patient number, which result from the very early premature stop of the trial, and the single‐center approach. These factors precluded any meaningful statistical analysis; thus, we provide only a descriptive analysis.

## 6 Conclusion

Patients with HF undergoing noncardiac surgery represent a population at high‐risk population of HF‐specific postoperative complications. Our pilot data suggest that pre‐ and postoperative nurse‐supported HF care is feasible and may have the potential to reduce postoperative HF‐related complications and the length of the hospital stay. Further clinical trials investigating risk reduction in this highly vulnerable surgical patient cohort are warranted.

## Author Contributions

Conceptualization: Birgit Assmus. Methodology: Birgit Assmus and Ester J. Herrmann. Software, SPSS (Version 27.0, SPSS Inc.) and R (Version 3.3.1, R Foundation for Statistical Computing, Vienna, Austria). Formal analysis: Ester J. Herrmann and Badrinarayanan Raghavan. Investigation: Birgit Assmus, Patrick Meybohm, and Ester J. Herrmann. Resources: LOEWE Center for Cell and Therapy Frankfurt funded by Hessisches Ministerium für Wissenschaft und Kunst, funding reference number III L 4‐518/17.004 (2010). Data Curation: Birgit Assmus, Patrick Meybohm, Ester J. Herrmann, Kathleen Mantzsch, and Meaza Tekeste. Writing–original draft preparation: Ester J. Herrmann. Writing–review and editing: Birgit Assmus, Ester J. Herrmann, and Patrick Meybohm. Visualization: Birgit Assmus. Supervision: Birgit Assmus. Project Administration: Birgit Assmus. Funding acquisition: Birgit Assmus. All authors have read and agreed to the published version of the manuscript.

## Ethics Statement

The study was conducted according to the guidelines of the Declaration of Helsinki and approved by the Ethics Committee of Goethe University, Frankfurt, Germany, (protocol number NCT03202329).

## Consent

Informed consent was obtained from all subjects involved in the study. Written informed consent has been obtained from the patients to publish this paper.

## Conflicts of Interest

B.A. reports having received consulting fees and an unrestricted research grant from St Jude Medical is now Abbott, and lecture fees from Abbott, AstraZeneca, Bayer, Boehringer Ingelheim, BMS, Edwards, Novartis, NovoNordisk, CSL Vifor and ZOLL. E.J.H. reports having received lecture fees from Bayer and CSL Vifor.

## Supporting information

Supporting information.

## Data Availability

The data on which this report are based are combined together and analyzed within the single‐center clinical trial (NCT03202329) and can be obtained by written request.
