# Supplementary material for: Randomized Pilot Trial of Pre‐ and Postoperative Heart Failure Nurse‐Supported Care in Heart Failure Patients Requiring Noncardiac Surgery—Feasibility and Results
Source: Clin Cardiol. 2024 Jun 24;47(6):e24304. doi: 10.1002/clc.24304 (PMC11194970; doi:10.1002/clc.24304)
Supplement: Supplementary file 1 — Supporting information. [file CLC-47-e24304-s001.docx]

**Supplementary Table S1: Secondary endpoint events at 90-days follow-up**

|  | **Control group**  **(n=11 of previously 18)** | **Intervention group**  **(n=11 of previously 16)** |
| --- | --- | --- |
| **Secondary endpoints** |  |  |
| **Duration of total hospital stay for non-cardiac surgery** (median, IQR, days) | 13.5 (7.3-18.5) | 14 (7.5-26) |
| **Duration on IMC/ICU for non-cardiac surgery** (median, IQR, days) | 0 (0-1.5) | 0 (0-0) |
| **Acute kidney injury** (n, %)  Stage 1  Stage 2  Stage 3 | 0, 0  0, 0  0, 0 | 0, 0  0, 0  0, 0 |
| **∆NT-proBNP** (bl. vs discharge, median, IQR, n, pg/ml) | 522 (213-3933) (n=11) | -473 (-3018-303) (n=8) |
| **∆Troponin** **T** (bl. vs. discharge, median, IQR, n, ng/ml) | 17 (5-36) (n=8) | -4 (-12-4) (n=3) |
| **Quality of life acc. to SF-12** (median, IQR) | 50 (50-81) (n=9) | 50 (50-75) (n=11) |
| **ΔQuality of life acc. to SF-12** (mean±SD) | 15±1.7 | 17±2.9 |

Abbreviations: IQR, interquartile range; IMC, intermediate care; ICU, intensive care unit; NT-proBNP, N-terminal fragment of pro-brain natriuretic

**Supplementary Table S2: Baseline medication**

| **Patient ID** | **Group** | **RAAS-inhibitor** | **BB** | **MRA** | **Diuretics** |
| --- | --- | --- | --- | --- | --- |
| 1 | Intervention | x | x | x | x |
| 2 | Intervention | x |  |  | x |
| 3 | Intervention | x | x |  | x |
| 4 | Intervention | x | x | x | x |
| 5 | Intervention |  |  | x | x |
| 6 | Intervention | x |  |  |  |
| 7 | Intervention | x | x | x | x |
| 8 | Intervention | x | x |  | x |
| 9 | Intervention | x | x |  | x |
| 10 | Intervention |  | x |  | x |
| 11 | Intervention | x | x |  | x |
| 12 | Intervention | x | x | x | x |
| 13 | Intervention |  | x |  | x |
| 14 | Intervention | x | x | x | x |
| 15 | Intervention | x | x | x | x |
| 16 | Intervention | x |  |  | x |
| 17 | Control | x | x |  | x |
| 18 | Control | x | x | x | x |
| 19 | Control | x | x |  | x |
| 20 | Control | x | x |  | x |
| 21 | Control | x | x |  | x |
| 22 | Control |  | x |  |  |
| 23 | Control | x |  |  | x |
| 24 | Control | x | x | x |  |
| 25 | Control |  | x |  | x |
| 26 | Control | x | x |  | x |
| 27 | Control | x | x | x | x |
| 28 | Control | x | x |  | x |
| 29 | Control |  | x |  | x |
| 30 | Control | x | x |  |  |
| 31 | Control |  | x |  |  |
| 32 | Control | x | x |  | x |
| 33 | Control | x | x |  | x |
| 34 | Control | x | x | x | x |

Abbreviations: RAAS-inhibitor=renin-angiotensin-aldosteron-inhibitor, bb= beta-blocker, MRA=mineralocorticoid receptor antagonist

**Supplementary Table S3: Secondary endpoints and exploratory analyses at 30-days follow-up**

|  |  | **Control group** | **Intervention group** |
| --- | --- | --- | --- |
| **Secondary endpoint events** | |  |  |
| **Duration of hospital stay for non-cardiac surgery** (median, IQR, days) | | 13.5 (7.3-18.5) | 14 (7.5-26) |
| **Duration on IMC/ICU for non-cardiac surgery** (median, IQR, days) | | 0 (0-1.5) | 0 (0-0) |
| **Number of pleural punctures** (n, %) | | 2 (1.1) | 0 (0) |
| **Number of pulmonary edema** (n, %) | | 0 (0) | 0 (0) |
| **Days of (non)-invasive ventilation** (median, IQR, days) | | 0 (0-0) | 0 (0-0) |
| **Days of antibiotic treatment for pneumonia** (median, IQR, days) | | 0 (0-0) | 0 (0-0) |
| **Days of inotropic therapy** (median, IQR, days) | | 0, (0-0) | 0, (0-0) |
| **Acute kidney injury** (n, %)  Stage 1  Stage 2  Stage 3 | | 1 (5.6)  2 (11.1)  0 (0) | 1 (6.3)  0 (0)  0 (0) |
| **Number of diuretic dose adjustments** (n, %) | | 43 (239) | 17 (106) |
| **Cardiac rhythm disorders** (n, %)  New onset of atrial fibrillation  Ventricular tachycardia  Implantation of pacemaker for bradycardia | | 1 (5.6)  1 (5.6)  0 (0) | 1 (6.3)  0 (0)  0 (0) |
| **∆NT-proBNP** (bl vs. 30d, median, IQR, n, pg/ml) | | 104 (-378-1063) (n=6) | 195 (-983-416) (n=12) |
| **∆Troponin T**  (bl vs. 30 d, median, IQR, n, pg/ml)) | | 6 (4-10) (n=5) | 0 (-15-4) (n=6) |
| **Death** (n, %) | | 1 (5.6)  (due to LAE) day 16 postop. | 0 (0) |
| **All rehospitalizations** (n, %) | | 3 (16.7) | 4 (25) |
| **Rehospitalizations for HF** (n, %) | | 2 (11.1) | 1 (6.3) |
| **Quality of life acc. to SF-12** (median, IQR) | | 50 (50-75) | 50 (25-66) |
| **ΔQuality of life acc. to SF-12** (mean±SD) | | 5.6±0.9 | 3.1±1.2 |

Abbreviations: IQR, interquartile range; IMC, intermediate care; ICU, intensive care unit; NT-proBNP, N-terminal fragment of pro-brain natriuretic

**Supplementary Table S4:** **SF-12 questionnaire**

| A | General health |
| --- | --- |
|  | **Are activities limited by health?** |
| B  C | Moderate activities limited?  Climbing several flights of stairs limited? |
|  | **Problems with work or daily activities due to physical health?** |
| D  E | Accomplished/achieved less?  Limited in kind of work or other activities? |
|  | **Problems with work or daily activities due to emotional problems?** |
| F  G | Accomplished (done) less?  Did work or activities less carefully than usual? |
|  |  |
| H | Pain interfering with normal work? |
|  | **Feeling** |
| I  J  K | Calm and peaceful  A lot of energy  Down-hearted and blue |
|  |  |
| L | How much has physical health or emotional problems interfered with social activities? |

**Supplementary Table S5:** **Results of SF-12** (mean±SD)

|  | A | B | C | D | E | F | G | H | I | J | K | L |
| --- | --- | --- | --- | --- | --- | --- | --- | --- | --- | --- | --- | --- |
| Control | 35±16 | 35±38 | 31±25 | 40±33 | 40±32 | 60±36 | 60±36 | 54±38 | 62±17 | 40±22 | 56±25 | 48±31 |
| Intervention | 27±15 | 14±31 | 11±21 | 38±31 | 39±31 | 48±39 | 50±40 | 48±42 | 59±23 | 32±27 | 59±35 | 64±31 |

Baseline:

30-day follow-up:

|  | A | B | C | D | E | F | G | H | I | J | K | L |
| --- | --- | --- | --- | --- | --- | --- | --- | --- | --- | --- | --- | --- |
| Control | 40±16 | 35±38 | 31±25 | 40±28 | 42±30 | 67±36 | 71±37 | 56±31 | 69±18 | 44±23 | 62±35 | 69±27 |
| Intervention | 30±15 | 32±37 | 25±26 | 32±36 | 39±38 | 43±45 | 50±44 | 55±31 | 59±29 | 36±32 | 68±33 | 57±36 |

90-days follow-up:

|  | A | B | C | D | E | F | G | H | I | J | K | L |
| --- | --- | --- | --- | --- | --- | --- | --- | --- | --- | --- | --- | --- |
| Control | 44±11 | 50±35 | 44±30 | 50±40 | 53±38 | 81±33 | 83±28 | 58±33 | 69±37 | 53±32 | 81±21 | 78±29 |
| Intervention | 48±13 | 50±45 | 36±39 | 52±31 | 43±32 | 68±30 | 59±29 | 73±36 | 77±21 | 52±21 | 68±23 | 66±34 |

**Supplementary Table S6:** **Results of quality-of-life questionnaire** (mean±SD)

|  | Control | | | Intervention | | |
| --- | --- | --- | --- | --- | --- | --- |
|  | Baseline | 30-day follow-up | 90-day-follow-up | Baseline | 30-day follow-up | 90-day-follow-up |
| Quality of Life | 35±16 | 40±16 | 44±11 | 27±15 | 30±15 | 48±13 |
| Physical Limitation | 37±31 | 37±30 | 49±35 | 25±49 | 32±34 | 45±36 |
| Emotional Limitation | 55±30 | 62±31 | 71±30 | 49±35 | 52±37 | 66±27 |
| Social Limitation | 48±31 | 69±27 | 78±29 | 64±31 | 57±36 | 66±34 |

**Supplementary Figure S1:** **Quality of life assessments using the SF12 questionnaire comparing baseline to 90-days follow-up.**

**A** Quality of life within the different dimensions of the SF 12 within the treatment groups. Comparison of baseline and 90-day follow-up results.

**B** Detailed assessment of individual questions for quality of life within the treatment groups. A = General health. (Are activities limited by health?) B Moderate activities limited? C = Climbing several flights of stairs limited? (Problems with work or daily activities due to physical health?) D = Accomplished/achieved less? E = Limited in kind of work or other activities? (Problems with work or daily activities due to emotional problems?) F = Accomplished (done) less? G = Did work or activities less carefully than usual? H = Pain interfering with normal work? (Feeling) I = Calm and peaceful. J = A lot of energy. K = Down-hearted and blue. L=How much has physical health or emotional problems interfered with social activities? Comparison of baseline and 30-days follow-up results.

**Supplementary Figure S2: Quality-of-life assessments using the SF12 questionnaire comparing baseline to 30-days follow-up results.**

Detailed assessment of individual questions pertaining to quality of life within the treatment groups. A = General health. (Are activities limited by health?) B = Moderate activities limited? C = Climbing several flights of stairs limited? (Problems with work or daily activities due to physical health?) D = Accomplished/achieved less? E = Limited in kind of work or other activities? (Problems with work or daily activities due to emotional problems?) F = Accomplished (done) less? G = Did work or activities less carefully than usual? H = Pain interfering with normal work? (Feeling) I = Calm and peaceful. J = A lot of energy. K = Down-hearted and blue. L = How much has physical health or emotional problems interfered with social activities?
